# Anthropogenic Disturbances and Invasion of *Mikania micrantha* Threaten *Rauvolfia serpentina* Populations in Nepal

**DOI:** 10.1002/ece3.72731

**Published:** 2025-12-22

**Authors:** Ajay Neupane, Bikram Jnawali, Suresh Kumar Ghimire

**Affiliations:** ^1^ Department of Botany, Mechi Multiple Campus Tribhuvan University Kathmandu Nepal; ^2^ Central Department of Botany Tribhuvan University Kathmandu Nepal; ^3^ CAS Key Laboratory for Plant Diversity and Biogeography of East Asia, Kunming Institute of Botany Chinese Academy of Sciences Kunming China

**Keywords:** conservation, endangered species, Indian snakeroot, invasive species, medicinal plants

## Abstract

*Rauvolfia serpentina* (L.) Benth. ex Kurz (family: Apocynaceae), a threatened medicinal plant in Tropical Asia, faces rapid depletion due to unsustainable harvesting, human‐mediated habitat alteration, and invasion of alien species. Lack of knowledge regarding its population status, ecology, and performance hinders effective species‐specific conservation management plans. Therefore, we studied the population density and structure of *R. serpentina* and assessed the variation in its life history traits (e.g., adult plant height, adult stem diameter, adult canopy diameter, number of branches, fruit circumference, dry fruit mass, and reproductive output), and regeneration status among populations at four sites differentially disturbed from human pressure and invasion of *Mikania micrantha* Kunth (family: Asteraceae)—highly disturbed, moderately disturbed, undisturbed, and previously disturbed—in the Jalthal forest in eastern Nepal. We conducted seed germination tests in a polyhouse to determine the seed germination potential and seedling growth performance on four different canopy types based on presence or absence of *Mikania micrantha* e.g., open without‐mikania, semi‐close without‐mikania, close with‐mikania and close without‐mikania. The highest density (2.53 ± 0.22 individuals 25 m^2^) was recorded in the undisturbed site. *R. serpentina* density greatly reduced with increasing disturbance. Compared to disturbed sites, undisturbed site showed high seedling recruitment and had adults with better growth performance and reproductive output. Habitat destruction and alteration, invasion of alien species, over‐harvesting, and poor seed viability are the probable reasons behind the reduced regeneration and low population density of *R. serpentina* in Jalthal forest. The results showed that *R. serpentina* was very sensitive to anthropogenic disturbances and the invasion of alien species, which contributed to the variation in density, life‐history traits, and regeneration potential in differentially disturbed sites. Furthermore, this species struggles to sustain and compete with others in disturbed habitats due to its low seed germination.

## Graphical abstract

This research article investigate the current population dynamics of a threatened medicinal plant *Rauvolfia serpentina* .in differentially disturbed region of tropical forest in eastern Nepal. The findings revealed the cumulative impacts of over exploitation and invasion of *Mikania micrantha* have negatively impacted the population dynamics of *R. serpentina* and due to its low seed germination rate, it is difficult for this species to sustain and compete with other species in disturbed habitats.

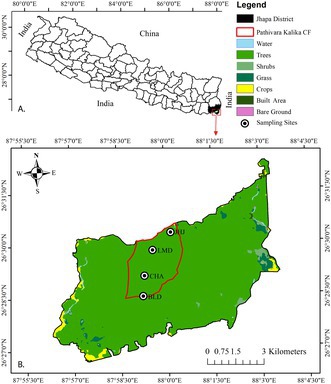

## 1 Introduction

Plants are facing multiple anthropogenic threats, such as habitat fragmentation and alteration, deforestation, unsustainable harvesting, invasion of alien species, overgrazing, forest fires, urbanization, pollution, and climate change, which may lead to rarity and endangerment (Convention on Biological Diversity 2009). In addition, the intrinsic characteristics of a plant species, such as habitat specificity, small population size, restricted geographical distribution, loss of competitive ability, and low investment in reproductive output, are also related to its rarity and endangerment (Primack 2010; Rabinowitz 1981). Anthropogenic disturbances may influence the evolution of life‐history traits (Tonnabel et al. 2018), population growth rate, recruitment dynamics (Chapagain et al. 2019), and the regeneration status of a plant species (Shrestha et al. 2016). The assessment of plant performance in relation to a variety of anthropogenic disturbance levels can be used to predict the response of species to altered habitat (Totland and Nyléhn 1998).

Over the last few decades, the use of medicinal plants has gone from subsistence collection to large‐scale commercial extraction due to high demand from growing natural product industries, which has increased the probability of over‐exploitation and endangerment of many species (Ghimire et al. 2007; Vasisht et al. 2016). One of such medicinal plant is *Rauvolfia serpentina* (L.) Benth. ex Kurz (family: Apocynaceae), which is a highly valued species, indigenous to the moist, deciduous forest of Tropical Asia, which includes, Cambodia, Bangladesh, East Pakistan, India, Indonesia, Jawa, Laccadive island, Laos, Lesser Sunda island, Malaya, Myanmar, Nepal, South‐central China, Sri Lanka, Thailand, and Vietnam (Dey and De 2010; Plants of the World Online 2024). In Nepal, it occurs widely in tropical and subtropical regions from east to west up to an elevation of 900 m (Department of Plant Resources 2007; Hara et al. 1979).

*Rauvolfia serpentina* , commonly known as “Indian snakeroot” (*Sarpagandha*, in Nepali), is an erect, small, evergreen, perennial semi‐shrub, that usually reaches heights of 20–55 cm however, sometimes up to 90 cm tall. It is mainly known for its roots, which contain approximately 30 alkaloids (Mittal et al. 2012) and has high demand in both domestic and international markets (Gaire et al. 2022; Mulliken and Crofton 2008). The roots of *R. serpentina* are used in Ayurveda, Unani, Homeopathy, and Siddha systems of medicine for the treatment of various diseases, such as cholera, diarrhea, dysentery, intestinal disorders, acute abdominal pain, asthma, hypochondriasis, high blood pressure, cardiac diseases, and various central nervous system disorders (Baral and Kurmi 2006; Choudhary 2003; Joshi and Joshi 2006; Phatak et al. 2017; Sharma 2004). Considering its commercial importance, the Government of Nepal nominates it as one of the thirty herbs of national priority for development, research, and cultivation (Department of Plant Resources 2012). Similarly, the National Medicinal Plants Board of India also listed *R. serpentina* as one of the “medicinal plants for development and cultivation on priority” (Maiti and Geetha 2013). However, due to overexploitation for commercial use, the population of *R. serpentina* is declining throughout its distribution range, leading to its inclusion in conservation priority lists (Mulliken and Crofton 2008). In Nepal, *R. serpentina* is designated “critically endangered” by the Conservation Assessment and Management Plan (CAMP) workshop and its trade is regulated under CITES (Table S2) (Bhattarai et al. 2002; Joshi et al. 2017; Mulliken and Crofton 2008). *R. serpentina* is also banned for export outside Nepal without processing (Department of Plant Resources 2012). In India, it is listed as “critically endangered” in the states of Maharastra, Chattisgarh, Andra Pradesh and “vulnerable” in Kerala, Orissa and Tamil Nadu (as cited in Sharma et al. 2022) and the trade of wild *R. serpentina* is prohibited (Maiti and Geetha 2013). Similarly, *R. serpentina* is listed “critically endangered” in Bangladesh (Khan et al. 2001) and in Vietnam (Ministry of Science and Technology (MOST) and Vietnam Academy of Science and Technology (VAST) 2007) Red Data Books.

Despite limited trade data, the roots of *R. serpentina* are extensively collected from the wild population for trade annually, contributing to the rapid depletion of this species in the wild (Department of Plant Resources 2013; Mulliken and Crofton 2008). The tropical and sub‐tropical forests of Nepal serve as one of the main sources of traded *R. serpentina* in local and international markets (Department of Plant Resources 2013). Particularly, the *R. serpentina* populations in the tropical forest at core Terai region of Nepal are heavily exploited and face grave extinction risk (Gaire et al. 2022). Illegal harvesting of Non‐ Timber Forest Products (NTFPs), disturbances, such as selective felling, encroachment, uncontrolled grazing, annual forest fires, lack of conservation awareness among local residents, and invasion of alien species have deteriorated the habitat condition and altered the species composition of tropical Sal ( *Shorea robusta* C.F.Gaertn.; family: Dipterocarpaceae) forest, thus suppressing the growth and regeneration of native species, such as *R. serpentina* (Rahman et al. 2009; Sharma et al. 2024; Srivastava et al. 2014). Furthermore, poor seed germination rate is one of the major causes of the decline of *R. serpentina* from its natural habitat (Dey and De 2010). In addition to the over‐exploitation and poor seed regeneration potentiality, habitat degradation by invasive plant species also appears to be an important threat to *R. serpentina* . Invasive species have been widely accounted for the cause of the decline and/or extinction of hundreds of native species (Dueñas et al. 2018; Ricciardi et al. 1998, 2017; Shrestha et al. 2024). *Mikania micrantha* Kunth (hereafter *Mikania*; family: Asteraceae) has been identified as one of the 100 worst invasive alien species and one of the top 10 weeds in the world (Lowe et al. 2000; Zhang et al. 2004). It is one of the most problematic invasive species in tropical forests and shrublands, particularly in Eastern and Central Nepal (Shrestha 2016; Tiwari et al. 2005). *Mikania* is known to decrease the density of native species (Kaur et al. 2012). *Mikania* not only competes with native species for resources but also grows in such a way that native species cannot sustain themselves in their natural habitat (Chen et al. 2024; Yin et al. 2020; Zhao et al. 2023). As *R. serpentina* and *Mikania* both prefer open areas and canopy gaps in the forest, the invasion of *Mikania* may directly hamper the existence of *R. serpentina* in the forest by competition and habitat alternation (Kunwar 2019; Sooraj et al. 2020; Zhang et al. 2004).

Prioritization of endangered plant species, such as *R. serpentina* for conservation requires a comprehensive understanding of their distribution pattern, population ecology, regeneration, and overall performance according to habitat conditions, including disturbance (Schemske et al. 1994; Singh et al. 2017; Watson et al. 1994). Despite the economic importance and high conservation value of *R. serpentina* , detailed studies describing its population ecology and performance in the wild are still lacking. This is problematic, as it is important to distinguish the causes and consequences of rarity and endangerment to effectively conserve rare and endangered species (Watson et al. 1994). Scientific information describing its responses (population density, structure, and regeneration) under different disturbance regimes is essential for the development of species‐specific management strategies (Ghimire et al. 2005; Ticktin 2004), which can be a stepping stone towards the conservation of *R. serpentina* in the wild. Thus, in this study, we set our objectives as (i) to analyze population structure and density of *R. serpentina*, (ii) to access its life‐history traits, and (iii) to examine regeneration status incorporating seed germination test along the disturbance gradient and intensity of *Mikania* invasion.

## 2 Material and Methods

### 2.1 Study Area

**FIGURE 1:**
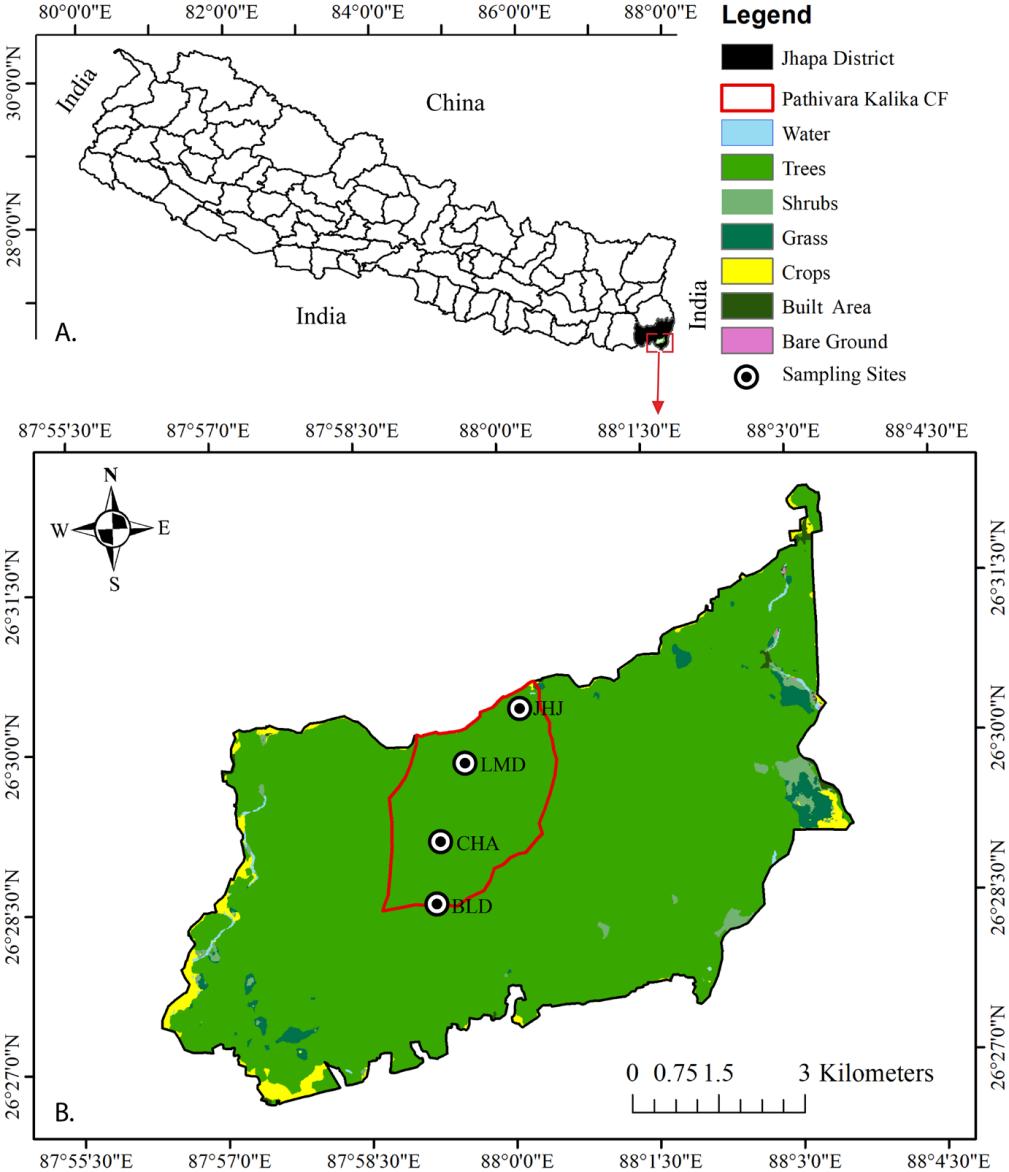
Map showing study area, (A) Nepal; (B) Jalthal forest area showing Pathivara Kalika Community Forest (CF) and sampling sites (BLD, Beldanda; CHA, Charali; JHJ, Jharjhar; LMD, Lamidanda).

The study was conducted in Jalthal forest (87° 55′ and 88° 03′ E/26° 27′ and 26° 32′ N), one of the largest Sal forest of Jhapa District, of Eastern Nepal (Figure 1). It covers 6100 ha (hectare) area in the southern part of the district and the forest elevation ranges from 62 to 129 m asl (meter above sea level) (Bhattarai 2017; Sharma et al. 2024). The climate is tropical monsoon type with three distinct seasons: dry and warm summer (March to mid May), humid and warm rainy (mid May to October) and dry and cool winter (mid November to February) (Bhattarai 2017). Jalthal forest is a unique tropical mixed‐broadleaved forest dominated by Sal ( *Shorea robusta* ) and is characterized by the presence of tree species belonging to subtropical forests, such as *Castanopsis indica* (Roxb. ex Lindl.) A.DC. (family: Fagaceae), *Schima wallichii* (DC.) Korth. (family: Theacecae) and *Madhuca longifolia* (L.) J.F. Macbr. (family: Sapotaceae), and some threatened plant species, such as *Senegalia catechu* (L.f.) P.J.H. Hurter & Mabb. (family: Fabaceae), *Bombax ceiba* L. (family: Malvaceae), *Cycas pectinata* Buch.‐Ham. (family: Cycadaceae), *Dalbergia latifolia* Roxb. (family: Fabaceae) and *Magnolia champaca* (L.) Baill. ex Pierre (family: Magnoliaceae) (Bhattarai 2017; Chaudhary et al. 2015). Despite its uniqueness and wealth of biodiversity, the forest is disturbed by a variety of causes, mainly, anthropogenic activities, invasion of alien species, and forest fires (Bhattarai 2017; Sharma et al. 2024). Although the forest is under the management of 22 Community Forest User Groups (CFUGs), existing management practices are lacking in terms of conserving the unique biodiversity and tackling the new threats like invasion of invasive species (Sharma et al. 2024). The invasion of alien species, particularly of *M. micrantha* and *Chromolaena odorata* (L.) R.M. King & H. Rob. (family: Asteraceae) was the most problematic in this forest, as over 30% of the forest is covered by these species (Regmi et al. 2023; Sharma et al. 2024).

### 2.2 Population Sampling

The sampling covered the Pathivara Kalika community forest (CF), the largest (740.96 ha) CF of the Jalthal Forest. Site selection was conducted in April 2017, with the help of locals and community forest officials. Participatory resource mapping was applied to identify the distribution of *R. serpentina* and its habitats in Pathivara Kalika CF (Kalibo and Medley 2007). The forest area with the distribution of *R. serpentina* population in the CF was stratified based on the anthropogenic disturbance level and the intensity of *Mikania* invasion. Based on information obtained from locals, community forest officials and preliminary field surveys, four sampling sites, namely Jharjhare, Lamidanda, Charali, and Beldanda (hereafter referred to as JHJ, LMD, CHA and BLD respectively) with the distribution of *R. serpentina* were determined for population sampling (Table 1).

**TABLE 1:** Classification of sampling sites based mainly on anthropogenic disturbance level and intensity of *Mikania* invasion.

Site	Trail distance from near village	Intensity of *Mikania* invasion	Anthropogenic disturbance level	Remarks
JHJ	< 1 km	Extensive (vegetation covered by *Mikania*)	Highly disturbed	Activities like clearance of the forest floor and extraction of NTFPs have severely affected the habitat quality.
LMD	1–2 km	Less extensive (less spread of *Mikania* compared to JHJ)	Moderately disturbed	Received infrequent and irregular human encroachment.
CHA	> 2 km	Negligible	Undisturbed	Faced less anthropogenic encroachment.
BLD	> 3 km	Extensive (vegetation was covered by *Mikania*)	Previously disturbed	It was the area where logging used to be done in the past and roads were extended, but at present, the area is protected under the community forest management system. However, due to the spread of invasive species, mainly *Mikania*, the previously disturbed site was still disturbed, and the vegetation condition was similar to that of the highly disturbed site.

Ecological sampling was conducted from June to July 2017 during the flowering and fruiting period of *R. serpentina* . A transect (ca. 1000 m) was determined in each site. Following the transect, 10 sample quadrats measuring 10 m × 10 m were defined randomly in each site. These quadrats were at least 50 m apart from each other and each were divided into four sub‐plots (5 m × 5 m) (Kala and Dubey 2012). In total, 160 subplots were studied to document the population status, and vegetative and reproductive characteristics, and associated vascular plant species of *R. serpentina* .

### 2.3 Data Collection

*Rauvolfia serpentina* individuals were categorized into four size classes based on their height and reproductive status (Table 2). In each subplot, individuals of different size classes were counted, and the data obtained were used to calculate the population structure and density. Each adult (vegetative and reproductive) individual occurring in the subplot was further measured/counted for its height, stem diameter, number of main branches, canopy diameter, and reproductive output (the number of buds, flowers, fruits, and scars were summed, averaged and calculated as total reproductive parts per individual) to assess the effects of anthropogenic disturbances on the life‐history traits of *R. serpentina* . Mature fruits were collected from reproductive adults from each site and were measured (circumference and dry fruit mass) in the laboratory. Data related to topographical, biophysical, and floristic variables were documented for each plot. We recorded, collected and identified associated vascular plant species from each 5 × 5 m plots, following methods from Polunin and Stainton (1984), and Shrestha et al. (2022). Voucher specimens were stored at the Tribhuvan University Central Herbarium (TUCH), Kathmandu, Nepal, and a full list of associated vascular plant species is provided in Table S3. Soil samples were collected from all four corners and the center of each quadrat at the depth of 10 cm after removing surface litter. These five soil samples were mixed thoroughly to collect single soil sample (ca. 500 g) from each plot. Collected soils samples were air dried in the shade, brought to the laboratory within 15 days of collection, and analyzed for pH, moisture, humus, and organic matter contents using standard method (Gupta 2001). The presence of animal droppings, grazing, trampling, harvesting, and fire was recorded in each subplot on a categorical scale of 0 (no disturbance) to 4 (high disturbance) as a measure of the disturbance variables (Kala and Dubey 2012). We conducted a questionnaire survey to determine trends in availability (present and past), harvesting practices, and parts used of *R. serpentina* among locals through semi‐structured interviews. Altogether, 50 individual interviews and two focus group discussions were done. The respondents were the regular forest goers (37), forest officials (eight), and local healers (five) aged between 30 to 67 years, who were familiar with the species.

**TABLE 2:** Size classes of *Rauvolfia serpentina* individuals based on plant height and presence or absence of reproductive parts.

Size class	Character considered	
	Height	Reproductive parts (flowers and/or fruits)
Seedling (Sd)	< 10 cm	Absent
Juvenile (Js)	10 < 20 cm	Absent
Adult vegetative (Adv)	> 20 cm	Absent
Adult reproductive (Adr)		Present

### 2.4 Seed Germination Test

Low seed germination is considered a constraint for the stability of *R. serpentina* in the wild (Dutta et al. 1963). Invasive species like *Mikania* are known to affect the germination potentiality and growth performance of native species (Ismail and Mah 1993; Kaur et al. 2012). So, to find out the seed germination potentiality and the probable effect of *Mikania* on it, a germination test was done from September 2017 to March 2018. For this experiment, four different *R. serpentina* canopy types were chosen based on presence or absence of *Mikania*: (i) Open without‐mikania (canopy cover < 25%, absence of *Mikania*); (ii) semi‐close without‐mikania (canopy cover 25%–75%, absence of *Mikania*); (iii) Close with‐mikania (canopy cover > 75%, canopy of *Mikania*) and (iv) Close without‐mikania (canopy cover > 75%, total absence of *Mikania*). Ripe fruits (*n* = 200, 50 per canopy type) were collected from each canopy type. The fruits were pulped manually to extract the seeds, washed in clean water and dried under shade. Seeds were subjected to the floating test (Dutta et al. 1963) and were soaked in water for 24 h before sowing in plastic grow bags (Department of Plant Resources 2013; Paul et al. 2008; Phatak et al. 2017). The heavy seeds that sank in the water were used for sowing and the soil used was also collected from the respected canopy type. Altogether, 160 heavy seeds (40 per canopy type) were sown in 56 polyethylene grow bags (3 seeds per grow bag at the depth of *ca*. 1 cm keeping 5 cm space between seeds) (Dutta et al. 1963). For each canopy type, grow bags containing 20 seeds out of 40 were kept in open canopy treatment and the rest of 20 seeds were kept in close canopy treatment. The close canopy treatment was maintained by placing the grow bags in the lower compartment of the cultivation rack (7 ft × 4 ft × 4.5 ft) and covering it with green plastic. For open canopy treatment, the grow bags were kept on the upper shelf of the cultivation rack with no coverage except the transparent plastic of polyhouse. This whole arrangement was done inside a polyhouse of eight feet in length and 5 ft in breadth. Polyhouse was made by using bamboo poles and transparent plastics. The average temperature recorded in the day in open canopy treatment was 27.78°C ± 1.02°C, whereas the average temperature recorded in the close canopy treatment was 24.23°C ± 0.98°C. In early weeks, the grow bags were covered by husks until the seedling appeared. The grow bags were constantly watered on the daily basis. The number of germinated seeds, and seedling height and number of leaves were recorded (up to 25th week) at weekly intervals for analyzing seed germination potentiality and seedling growth performance, respectively.

### 2.5 Data Analysis

Both the parametric and non‐parametric statistical tests were performed to analyze the data. Non‐parametric tests were used when the data (even after transformation) did not meet normality and homogeneity of variance. Parametric one‐way Analysis of Variance (ANOVA) and non‐parametric Kruskal–Wallis test were conducted to analyze differences in specific variables among sampling sites. Multiple comparisons for various variables between different sites were performed using Mann–Whitney U tests. The variables related to disturbance (e.g., animal droppings, grazing, trampling, harvesting, and fire) were subjected to dimension reduction using principal component analysis (PCA). PCA is a multivariate statistical technique used to summarize the information content in several correlated variables into a smaller set of uncorrelated variables (principal components) (Jolliffe 2002).

Density and population structure (the relative proportions of seedling, juvenile, adult‐vegetative and adult‐reproductive) of *R. serpentina* were analyzed for each sampling site. The future regeneration status of a plant species can be predicted from the relative proportions of seedlings, juveniles, and adults in the total population (Bharali et al. 2012). The difference in rejuvenation (expressed as the sum of seedling and juvenile density divided by the density of reproductive adults) (Endels et al. 2007) among the four sampling sites was calculated to estimate in situ regeneration. Additionally, percentage seed germination and seedling growth were calculated for the four canopy types to estimate ex‐situ regeneration. Variation in specific size class density and overall density of *R. serpentina* among the four sampling sites were compared using non‐parametric Kruskal–Wallis test. Similar analysis was applied to compare the population structure of *R. serpentina* among the sites. Both parametric and non‐parametric analyses were used to compare the vegetative (e.g., adult plant height, adult stem diameter, adult canopy diameter and number of main branches) and reproductive (e.g., dry fruit mass, fruit circumference and total reproductive output) traits among the four sites.

Furthermore, the relationships between explanatory (biophysical) and dependent variables (plant density, number of main branches, adult plant height, adult stem diameter, adult canopy diameter, fruit dry mass, fruit circumference and total reproductive output) were analyzed using multiple regressions. Forward selection method was adopted to identify important explanatory biophysical variables for each dependent variable. Finally, to explore the relationship between *R. serpentina* and its environment, we performed a redundancy analysis (RDA) to assess how different environmental factors influence the abundance of vascular plant species associated with *R. serpentina* at various sampling sites. RDA is a linear method of constrained ordination, which models a set of response variables (species abundance) as a function of a set of explanatory variables (environmental factors), providing a measure of the explained variance and the strength of the relationship (Šmilauer and Lepš 2014). All the statistical analyses were performed using R 4.3.2 (R Core Team 2024) using the vegan package for multivariate analysis (Oksanen et al. 2024).

## 3 Results

### 3.1 Habitat Characteristics

The four sampling sites experienced differential level of disturbance (Table S1). The variables related to disturbance (e.g., animal droppings, grazing, trampling, harvesting, and fire) after dimension reduction resulted into two components (Table 3). Animal droppings, grazing, trampling, and harvesting were highly correlated with component one, whereas fire was correlated with component two. The first component (explaining 46.88% of variance) was therefore termed as harvesting and grazing disturbance and second component (explaining 25.59% of variance) as fire disturbance. Overall variance explained by these components was 72.48%.

**TABLE 3:** Rotated component matrix extracted by principal component analysis method. Rotation method: Varimax with Kaiser Normalization (Rotation converged in three iterations).

Variables	Component	
	1	2
Animal droppings	0.64	0.03
Grazing	0.84	0.28
Trampling	0.84	−0.09
Harvesting	0.72	0.49
Fire	0.03	0.98

The plots at four sampling sites differed in almost all biophysical variables studied (Table 4). *R. serpentina* occurred both in disturbed and undisturbed sites but its density greatly reduced with increasing disturbance. It mostly preferred areas with semi‐close tree and shrub canopy cover, where cover of forbs and grasses was low with sufficient bare‐ground (Table 4). Tree cover was highest in highly disturbed site and lowest in previously disturbed site. However, the shrub cover was highest in previously disturbed site, but was lowest in highly disturbed site. The tree cover and shrub cover of undisturbed site was intermediate between highly disturbed and previously disturbed sites (Table 4).

**TABLE 4:** Biophysical variables (mean ± SE) recorded in four sampling sites with varying amount of anthropological disturbance in Jalthal Forest*.

Biophysical variables	JHJ (highly disturbed)	LMD (moderately disturbed)	CHA (undisturbed)	BLD (previously disturbed)	*χ* ^2^	df	*p*	Overall mean
Elevation (m)	84.70 ± 0.19^a^	102.10 ± 1.24^b^	104.80 ± 0.47^b^	104.80 ± 0.75^b^	91.03	3	< 0.001	99.05 ± 0.76
Tree cover (%)	43.75 ± 4.50^a^	37.50 ± 4.24^a^	39.25 ± 2.92^a^	9.5 ± 1.13^b^	58.87	3	< 0.001	32.50 ± 0.35
Shrub cover (%)	44.25 ± 2.52^a^	47.63 ± 2.86^a^	58.63 ± 1.99^c^	68.13 ± 3.80^b^	50.77	3	< 0.001	54.66 ± 1.60
Forbs cover (%)	36.50 ± 1.48^a^	48.25 ± 2.05^b^	46.75 ± 1.17^b^	39.75 ± 2.59^a^	27.01	3	< 0.001	42.81 ± 1.02
Grass cover (%)	19.00 ± 1.28^a^	16.00 ± 1.33^a^	9.38 ± 0.74^c^	12.0 ± 0.89^b^	39.25	3	< 0.001	14.09 ± 0.61
Litter depth (cm)	10.22 ± 0.52^a^	9.85 ± 0.68^a^	7.15 ± 0.40^b^	10.80 ± 0.97^a^	17.08	3	0.001	9.51 ± 0.35
Litter cover (%)	22.80 ± 1.99^a^	19.45 ± 1.52^ab^	7.48 ± 0.56^c^	15.38 ± 1.69^b^	36.71	3	< 0.001	16.28 ± 0.89
Bryophytes cover on soil (%)	0.60 ± 0.13^a^	1.30 ± 0.13^b^	0.80 ± 0.26^a^	1.10 ± 0.17^b^	20.90	3	< 0.001	0.95 ± 0.09
Lichen cover on soil (%)	0.30 ± 0.07^a^	0.30 ± 0.07^a^	0.25 ± 0.03^b^	0.45 ± 0.08^a^	19.06	3	< 0.001	0.27 ± 0.04
Scree cover (%)	5.70 ± 0.48^a^	3.25 ± 0.36^bc^	3.48 ± 0.35^b^	2.33 ± 0.25^c^	37.77	3	< 0.001	3.69 ± 0.21
Bare‐ground (%)	15.10 ± 1.25^a^	11.45 ± 1.52^b^	32.10 ± 1.66^c^	29.00 ± 2.19^c^	66.41	3	< 0.001	21.91 ± 1.09
Bryophytes below vascular plant (%)	8.90 ± 1.33^a^	10.70 ± 1.08^a^	9.45 ± 0.99^c^	5.45 ± 0.85^b^	35.76	3	< 0.001	8.94 ± 0.50
Lichen below vascular plant (%)	7.10 ± 0.77^a^	11.30 ± 1.05^b^	10.53 ± 0.76^b^	7.65 ± 0.99^a^	14.37	3	0.002	9.14 ± 0.47
Soil pH	5.15 ± 0.02^a^	5.23 ± 0.03^ac^	5.68 ± 0.15^b^	5.24 ± 0.03^c^	8.90	3	0.031	5.33 ± 0.07
Soil moisture (%)	9.86 ± 2.19^a^	10.54 ± 0.53^a^	10.91 ± 0.33^a^	10.77 ± 0.42^a^	0.79	3	0.852	10.52 ± 0.51
Soil humus (%)	2.13 ± 0.12^a^	2.55 ± 0.48^a^	1.44 ± 0.43^a^	3.24 ± 0.57^a^	7.21	3	0.066	2.34 ± 0.25
Soil organic matter (%)	2.50 ± 0.06^a^	1.98 ± 0.10^b^	1.88 ± 0.04^c^	2.06 ± 0.05^d^	10.42	3	0.015	1.97 ± 0.12

*Note:* *χ* ^2^ and *p* values based on Kruskal–Wallis test. Values associated with same superscript letter are not statistically significant (multiple comparisons based on Mann–Whitney *U* test).

* Total sample size = 40 (10 plots per disturbance category).

Physico‐chemical analysis of soil collected from the sampling sites showed that *R. serpentina* preferred slightly acidic soil, with lower soil moisture, humus and organic matter contents. Overall, edaphic factors remained same for four sampling sites, except soil pH and soil organic matter (Table 4).

### 3.2 Ordination of Associated Species and Environmental Predictors

**FIGURE 2:**
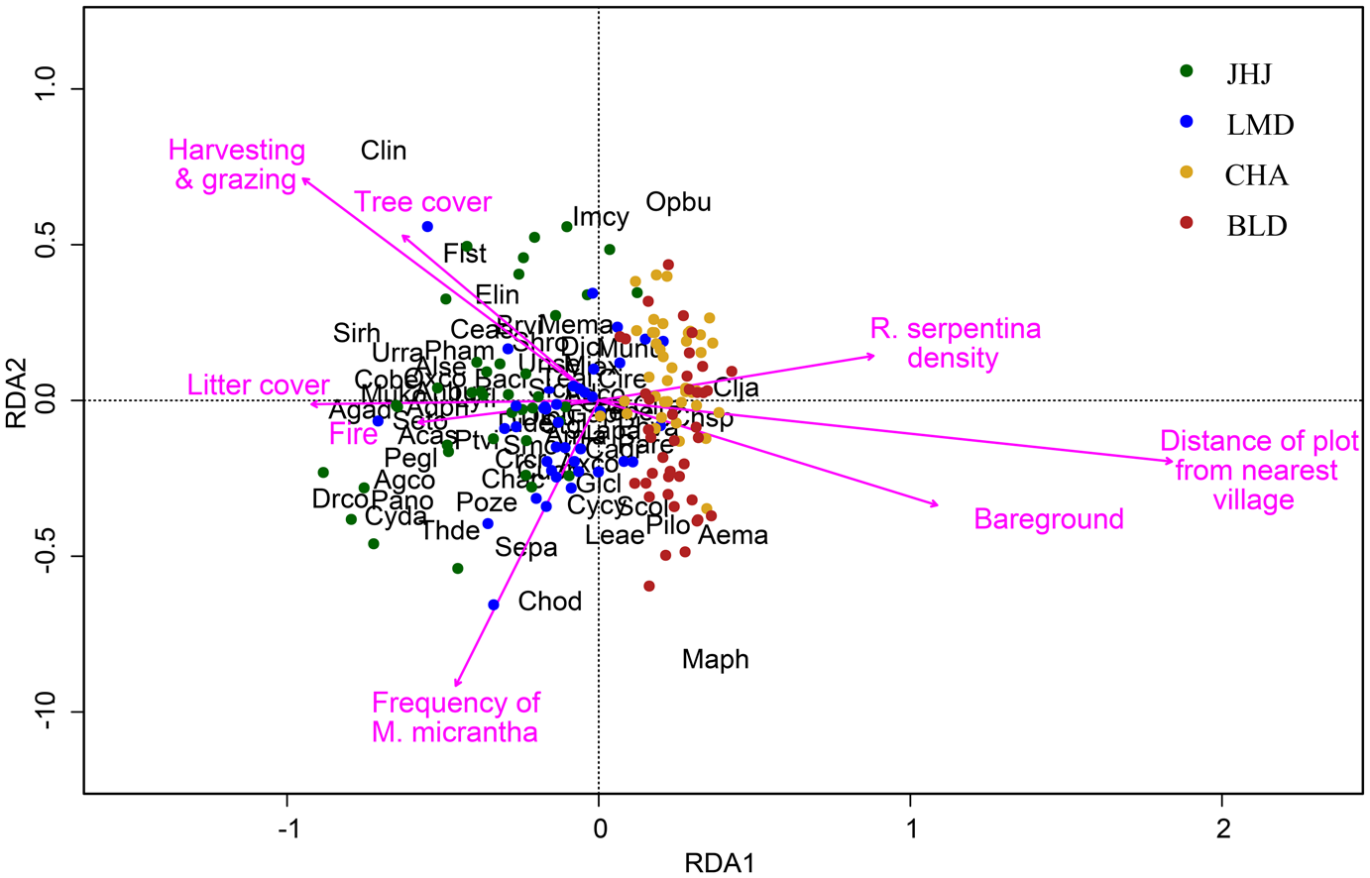
RDA biplot for associated plant species, biophysical variables, and one hundred twenty 5 m × 5 m plots in Jalthal forest. Full scientific names with four‐letter codes are available in Table S3.

Our redundancy analysis (RDA) showed that the main predictors of species identity and abundance in our 10 m × 10 m plots were cumulative disturbance, *R. serpentina* density, tree cover, bare ground, litter cover, distance of plot from the nearest village, and the frequency of *Mikania* (Figure 2). Of these, litter cover, fire, bare ground, and distance of plot from the nearest village were primarily associated with the first axis (RDA1), while the remaining factors loaded to the second axis (RDA2). The density of *R. serpentina* exhibited a near‐perfect inverse relationship with disturbance (harvesting, grazing, and fire) but was positively correlated with bare ground and distance of plot from the nearest village. Plots from CHA and BLD were clustered around areas with high *R. serpentina* density, while LMD and JHJ plots clustered near the disturbance vector with low *R. serpentina* density (Table 5, Figure 2).

**TABLE 5:** Density (number of individuals per 25 m^2^ area) and rejuvenation pattern of *Rauvolfia serpentina* in four differentially disturbed sites in Jalthal forest. Data shown are mean ± S.E. and statistical difference based on Kruskal–Wallis test*.

	JHJ (Highly disturbed)	LMD (Moderately disturbed)	CHA (Undisturbed)	BLD (Previously disturbed)	*χ* ^2^	df	*p*
Seedling	0.05 ± 0.05^a^	0.05 ± 0.04^a^	0.58 ± 0.13^b^	0.13 ± 0.06^a^	29.24	3	< 0.001
Juvenile	0.15 ± 0.07^a^	0.28 ± 0.09^a^	0.6 ± 0.11^b^	0.35 ± 0.08^ab^	15.61	3	0.001
Adult‐vegetative	0.08 ± 0.04^a^	0.08 ± 0.04^a^	0.50 ± 0.10^b^	0.08 ± 0.04^a^	30.42	3	< 0.001
Adult‐reproductive	0.53 ± 0.11^a^	1.00 ± 0.14^b^	0.85 ± 0.13^b^	1.15 ± 0.16^b^	9.97	3	0.019
Total population	0.80 ± 0.14^a^	1.4 ± 0.19^b^	2.53 ± 0.22^c^	1.70 ± 0.2^b^	33.52	3	< 0.001
Rejuvenation	0.10 ± 0.06^a^	0.23 ± 0.07^ab^	0.55 ± 0.14^c^	0.38 ± 0.10^bc^	13.46	3	0.004

*Note:* *χ* ^2^ and *p* values based on Kruskal–Wallis test. Values associated with same superscript letter were not statistically significant (multiple comparisons based on Mann–Whitney *U* tests).

* Total sample size = 40 (10 plots per disturbance category).

### 3.3 Population Density and Structure

We observed a significant difference in the total population density of *R. serpentina* among four sampling sites (Table 5). Additionally, we found significant differences in the observed size class distributions among these sites. Among the four sites, the undisturbed site had a significantly (*χ* ^2^ = 33.52, df = 3, *p* < 0.001) high density of *R. serpentina* . The population from undisturbed site also accounted for significantly higher density of seedlings, juveniles, and adult‐vegetative individuals (Table 5).

**FIGURE 3:**
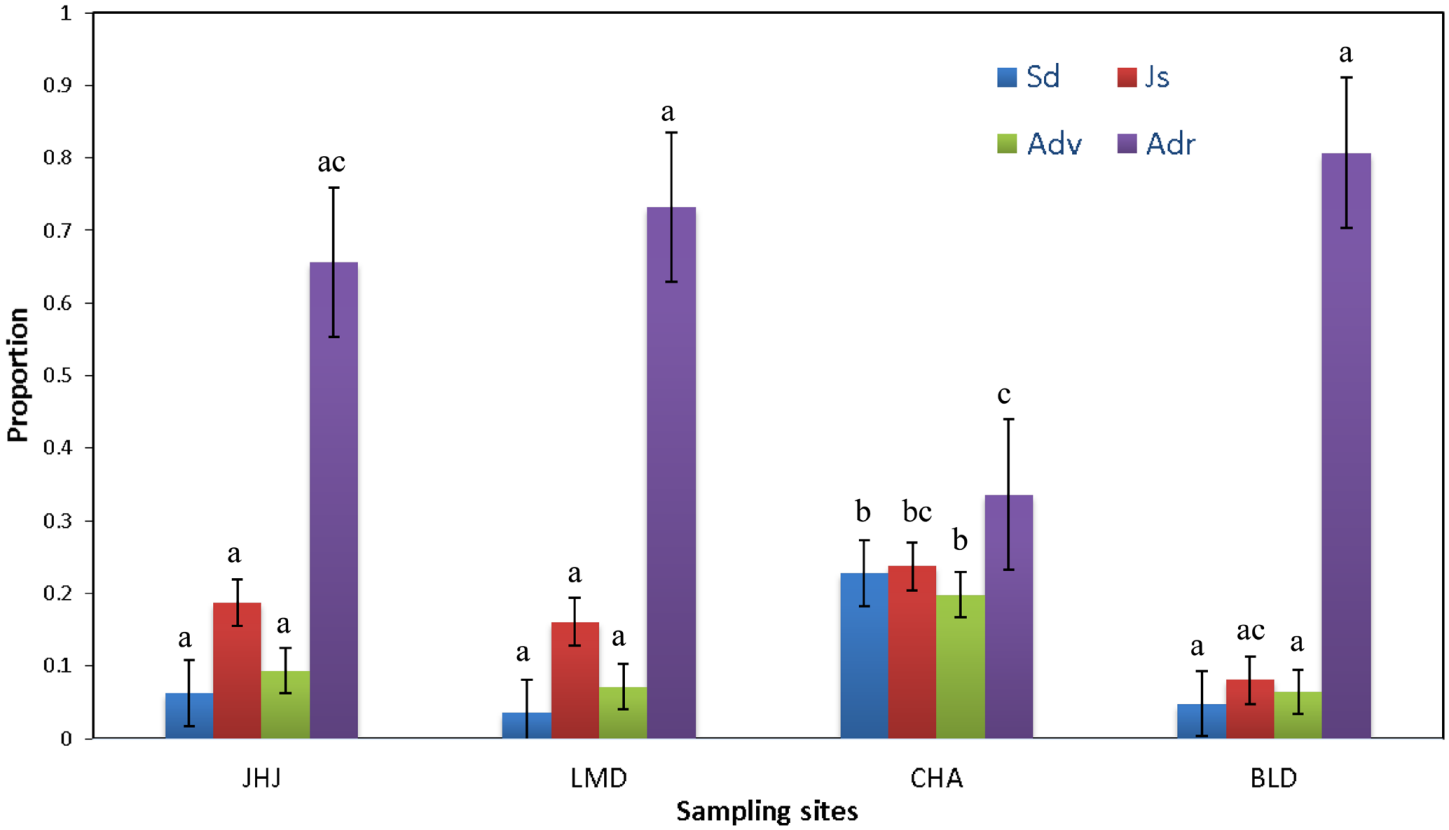
Population structure of *Rauvolfia serpentina* in Jalthal forest, eastern Nepal (Adr, adult reproductive; Adv, adult vegetative; Jv, juveniles; Sd, seedlings). Bars with same letter for each stage class among populations do not vary significantly at *p* = 0.05.

Overall, all four sites displayed similar population structures, with more adult than seedling and juvenile (Figure 3). Notably, the undisturbed site had a significantly higher proportion of seedlings, juveniles, and adult‐vegetative individuals compared to other sites.

### 3.4 Variation in Life–History Traits

The population of *R. serpentina* in the undisturbed site showed significantly higher growth performance compared to highly disturbed site, in terms of adult canopy diameter, number of main branches and reproductive output per individual (Table 6). However, in general, adult plant height, adult stem diameter, dry fruit mass and fruit circumference did not differ significantly among the sampling sites, though the values for all these traits were smaller in the highly disturbed site compared to other sites (Table 6). Hence, individuals of *R. serpentina* in the highly disturbed site exhibited low growth performance in terms of almost all life‐history traits studied.

**TABLE 6:** Life‐history traits of *Rauvolfia serpentina* in four sites. Data shown are mean ± S.E.

Life‐history traits	JHJ (Highly disturbed)	LMD (Moderately disturbed)	CHA (Undisturbed)	BLD (Previously disturbed)	Overall mean	*F*/*χ* ^2^ *	df	*p*
Adult plant height (cm)	10.90 ± 1.20	16.3 ± 1.96	14.11 ± 1.47	15.63 ± 1.77	14.23 ± 0.91	2.39	3	0.71
Adult stem diameter (cm)	0.16 ± 0.03	0.23 ± 0.03	0.23 ± 0.02	0.22 ± 0.02	0.21 ± 0.01	2.09	3	0.10
Adult canopy diameter (cm)	3.73 ± 0.72^a^	6.27 ± 0.86^b^	6.92 ± 0.87^b^	6.10 ± 0.90^b^	5.10 ± 0.91	2.10	3	0.03
No. of main branch	1.20 ± 0.09^a^	1.18 ± 0.07^a^	1.63 ± 0.11^b^	1.13 ± 0.05^a^	1.28 ± 0.04	10	3	< 0.001
Dry Fruit mass (gm)	0.16 ± 0.02	0.19 ± 0.01	0.21 ± 0.01	0.19 ± 0.02	0.19 ± 0.01	2.30	3	0.09
Fruit circumference (cm)	2.09 ± 0.04	2.16 ± 0.03	2.18 ± 0.04	2.20 ± 0.06	2.16 ± 0.02	1.37	3	0.27
Reproductive output per individual	8.41 ± 1.99^a^	15.41 ± 2.38^b^	16.15 ± 2.51^b^	14.88 ± 2.00^b^	11.51 ± 0.90	9.71	3	0.021

*Note:* Values associated with same superscript letter are not statistically significant (*p* > 0.05) (multiple comparisons based on Mann–Whitney *U* tests).

* Denotes test statistics values of non‐parametric (Kruskal–Wallis) test for the variables, which were non‐normal (even after transformation); and parametric (ANOVA) test for the variables, which were log normal.

### 3.5 Percentage Seed Germination and Seedling Growth

Overall percentage germination of cultivated *R. serpentina* seeds were found out to be 20% and seeds collected from close with‐mikania canopy type showed the lowest percentage germination (15%) compared to other canopy types (Table 7). We found differential effects of open and close canopy treatments on germination rate depending upon the canopy type from where seeds were collected (Table 7). However, comparing percentage germination based on canopy treatment, we found higher seed germination in open canopy treatment than in close canopy treatment (Table 7). These results indicated that *R. serpentina* seeds favor open canopy to germinate.

**TABLE 7:** Percentage germination of *Rauvolfia serpentina* seeds collected from four canopy types and grown in open and close canopy treatments in polyhouse.

Canopy types	% seed germination in open canopy treatment	% seed germination in close canopy treatment	Total % seed germination per site
Open without‐mikania	35	10	22.5
Semi‐close without‐mikania	25	20	22.5
Close with‐mikania	20	10	15
Close without‐mikania	25	15	20
Overall % seed germination	26.25	13.75	20

**FIGURE 4:**
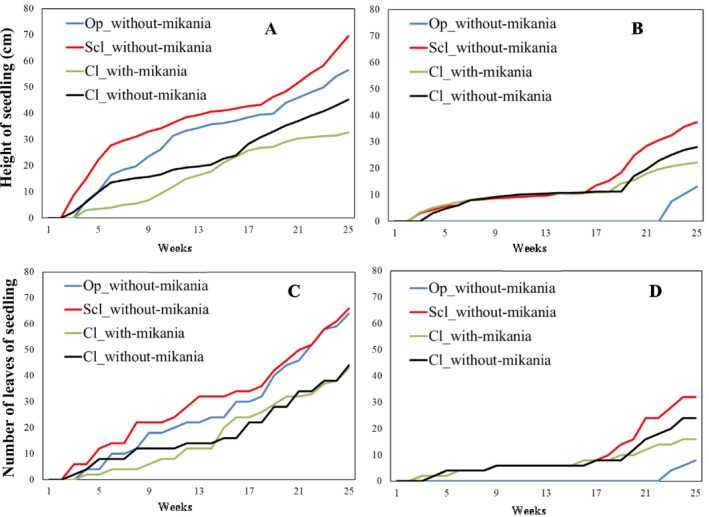
Comparison of seedling growth (based on height and numbers of leaves of seedling) of *Rauvolfia serpentina* germinated in open and close canopy treatment in polyhouse. (A = weekly height of seedling in open canopy treatment, B = weekly height of seedling in close canopy treatment, C = weekly number of leaves of seedling in open canopy treatment, D = weekly number of leaves of seedling in close canopy treatment; Op_without‐mikania = Open without‐mikania canopy type, Scl_without‐mikania = semiclose without‐mikania canopy type; Cl_with‐mikania = Close with‐mikania canopy type, and Cl_without‐mikania = Close without‐mikania canopy type).

Similarly, comparing the seedling growth performance between two canopy treatments, significantly high growth performance was found in seedling germinated and grown in open canopy treatment than those under close canopy treatment in polyhouse (Table 8). Seedlings from semi‐close without‐mikania canopy type showed higher growth performance in both canopy treatment compared to other canopy types (Figure 4). In general, the seedlings belonging to close with‐mikania canopy type showed low growth performance compared to close without‐mikania canopy type (Table 8, Figure 4).

**TABLE 8:** Height and number of leaves of *Rauvolfia serpentina* seedlings up to 25th weeks after germination in close and open canopy treatment in polyhouse. Data shown are mean ± S.E.

Canopy types	Cumulative height (cm)		Cumulative number of leaves	
	Open canopy treatment	Close canopy treatment	Open canopy treatment	Close canopy treatment
Open without‐mikania	29.86 ± 3.42	1.24 ± 0.70	25.72 ± 3.90	0.72 ± 0.41
Semi‐close without‐mikania	36.75 ± 3.56	14.01 ± 2.22	29.8 ± 3.75	10.24 ± 1.97
Close with‐mikania	16.35 ± 2.38	10.98 ± 1.22	16.48 ± 2.81	7.04 ± 0.93
Close without‐mikania	22.02 ± 2.67	11.46 ± 1.589	17.6 ± 2.52	8.16 ± 1.39

### 3.6 Availability and Harvesting Approach

*Rauvolfia serpentina* is one of the important medicinal plant species harvested by the locals of the Jalthal forest area. When asked about the availability of *R. serpentina*, the majority of respondents (84%) reported that availability has decreased by more than 50% over the last decade, which was later validated by focus group discussions. Conversely, few respondents (12%) stated that it is frequently available inside the forest. The remaining 4% said they have not assessed availability, but admitted the difficulty in finding the species nowadays. The root is the most commonly harvested and utilized part, although a few respondents (8%) stated to harvest the leaves as well for various other ailments, such as eye infection. Locals of the Jalthal forest harvest *R. serpentina* whenever and wherever they encounter it, for both immediate and future use. Unfortunately, the current harvesting practice is unsustainable, as the entire plant is uprooted to obtain its roots, leaving other parts wasted and also killing the individual. However, a minority (12%) of respondents mentioned leaving a portion of the root with the plant during harvesting, aiming to promote sustainability.

## 4 Discussion

### 4.1 Ordination of Associated Species

In our study, we found species that both coexisted with *R. serpentina* , often appearing together in the same areas, and showed a positive correlation with its density. These species, which met both criteria, included *Clerodendrum japonicum* (Thunb.) Sweet (Clja), *Murdannia nudiflora* (L.) Brenan (Munu), *Cissus repens* Lam. (Cire), *Hellenia speciosa* (J.Koenig) S.R.Dutta (Chsp), *Panicum repens* L. (Pare), and *Ampelocissus latifolia* (Roxb.) Planch. (Amla). Furthermore, our RDA analysis revealed that the JHJ and LMD sites differed significantly in environmental factors and associated species, in contrast to both the CHA and BLD sites. The species most commonly associated with *R. serpentina* at JHJ and LMD plots were *Oxalis corniculata* L. (Oxco), *Pteris vittata* L. (Ptvi), *Commelina benghalensis* L. (Cobe), *Chrysopogon aciculatus* (Retz.) Trin. (Chac), *Pouzolzia zeylanica* (L.) Benn. (Poze), *Alternanthera sessilis* (L.) DC. (Alse), and *Sida cordata* (Burm.f.) Borss.Waalk. (Sico) (Figure 2, Table S3). The prevalence of these species around the JHJ and LMD sites, whether due to their tolerance of denser *Mikania* canopies or higher disturbance levels, cannot be determined from our study.

### 4.2 Variation in Population Density and Structure

The deteriorated habitat condition due to anthropogenic disturbances and the invasion of alien species has negatively impacted the population density, and structure of *R. serpentina* in Jalthal forest. Habitat quality significantly influences the distribution of plant species, affecting their population density, reproduction, and regeneration (Łomnicki 1980; Pulliam and Danielson 1991). Anthropogenic disturbance can directly and/or indirectly impact the population dynamics of rare and endangered plant species like *R. serpentina* (Dutta et al. 1963; Pavlovic 1994). Direct disturbances, such as harvesting roots, directly harm individuals, while indirect disturbances like invasion of alien species, fire, grazing, and trampling indirectly affects the survival of the *R. serpentina* population in Jalthal forest. Harvesting and grazing disturbances significantly affected the adult plant density in the study area (as also discussed in Ghimire 2008) (Table S2). Harvesting of underground parts, such as rhizomes, roots, and bulbs has been reported to be unsustainable, even at a low level and sustainable harvesting of such parts presents a particular challenge (Ghimire et al. 2007). The unsustainable harvesting of *R. serpentina* roots has significantly reduced overall population density in the disturbed region, threatening its long‐term persistence in Jalthal forest (Ghimire 2008; Ghimire et al. 2005; Ghimire 2008; Kunwar 2019; Nair et al. 2014; Oosttermeijer et al. 1998; Ticktin 2004).

Populations with a high number of reproductive adults are known to withstand environmental stochastic forces (Endels et al. 2007; Hartnett and Bazzaz 1985; Li et al. 2013). A large population with more seedlings and juveniles in the undisturbed site indicates successful regeneration, and the population of *R. serpentina* from the undisturbed site could withstand different stochastic factors of the environment (Saikia and Khan 2013; Saxena and Singh 1984). In general, the population structure of *R. serpentina* in the study sites exhibited a J‐shaped curve which showed that the contribution of adults to the total population was highest followed by juveniles and seedlings. This indicates decreasing population of *R. serpentina* in the Jalthal Forest and signifies the unsustainability of the species in the future if the habitat destruction activities remain unchanged (Saikia and Khan 2013).

### 4.3 Variation in Life—History Traits

Life‐history traits of *R. serpentina* varied differentially across four sampled sites. Adult individuals from the highly disturbed site exhibited the lowest values for all life‐history traits, indicating a direct impact of disturbance on plant vigor and performance (as discussed in Nair et al. 2014). This decline may be the result of damage caused by local people collecting vegetables, fodder, fuelwood, and NTFPs, as well as activities, such as bush cleaning and floor clearing in the highly disturbed site (Saikia and Khan 2013; Sharma et al. 2021). Additionally, competition between *R. serpentina* and invasive alien species likely contributes to the low growth performance (Chen et al. 2024; Poudyal 2013; Yin et al. 2020; Zhao et al. 2023).

In contrast, adult individuals from the undisturbed site displayed higher values for life‐history traits compared to those from the highly disturbed site (Table 6). This difference may be attributed to the availability of suitable habitat conditions and the absence of invasive species in undisturbed sites. Disturbance is known to play a crucial role in limiting plant growth (Nair et al. 2014). Adults from the highly disturbed site were shorter, less branched and had weaker stem with smaller canopies than those from the undisturbed site. These individual typically produce terminal inflorescence with lesser number of flowers, which subsequently produced a higher proportion of lighter non‐viable seeds (Table 6). Conversely, adult plants from the undisturbed site exhibited the highest vegetative vigor. They were taller and shrubbier with much branched, stouter stem and larger canopies than the adults from the highly disturbed site. These individuals produced numerous inflorescences, producing a greater number of heavier fruits (as discussed in Given 1975). Hence, adults from the undisturbed site appeared more competitive and more resilient than those from disturbed sites (Table 6).

### 4.4 Regeneration Status

Successful regeneration is arguably the most crucial step for long‐term persistence of a species (Saikia and Khan 2013). Rejuvenation study exhibited the highest value in the undisturbed site, indicating greater stability of the population compared to other sites (Table 5). The presence of a sufficient proportion of seedlings, juveniles, and reproductive‐adults in the undisturbed site indicates successful regeneration and the potential for long‐term persistence of *R. serpentina* (Saikia and Khan 2013; Saxena and Singh 1984). However, all other three sites showed lower values for rejuvenation, indicating uncertainty of *R. serpentina* population and lesser probability of long‐term persistence (Saikia and Khan 2013). Notably, the highly disturbed site had the lowest rejuvenation score among all four sites. This suggests the *R. serpentina* population in that site faces a serious problem due to anthropogenic disturbance and invasion of *Mikania*. If the condition remains unchanged, the population of *R. serpentina* in the site may not persist in near future. Overall, anthropogenic disturbances have significantly impacted the regeneration of *R. serpentina* (as also discussed by Barik et al. 1996).

### 4.5 Seed Germination Potentiality and Growth Performance of Seedling

Although the seeds of *R. serpentina* appear morphologically normal, they exhibited a very poor germination rate (Dutta et al. 1963; Phatak et al. 2017). We determined an overall seed germination of 20% for *R. serpentina* , which aligns to the findings of Panwar and Guru (2012) (20.47%) and Muneshwar (2015) (20.67%), but is higher than the rates reported by Phatak et al. (2017) (16.67%) and Hussain and Jha (2014) (11.27% in unsoaked seeds). Under the open canopy treatment alone, the seed germination was found to be 26.25%, consistent with the findings of Paul et al. (2008) (26%) (Table 7). However, our overall seed germination rate of *R. serpentina* was still lower than that reported by Dutta et al. (1963), (38.2%), Sharma and Tyagi (2015) (28%), Malik and Swain (2018) (29.08%) and Tiwari et al. (2021) (39.33%). Notably, these above‐mentioned researchers, did not incorporate canopy treatment in their methodologies.

*Rauvolfia serpentina* is generally considered a partial‐shade adapted plant (Trivedi and Kumari 2009), and previous research suggested no significant effect of artificial shading on its growth (Samanhudi et al. 2017). However, our study revealed a significant difference in percentage seed germination and seedling growth performance between open and close canopy treatments. The percentage germination of seeds and growth performance of seedling from open without‐mikania canopy type clearly showed the effect of micro‐climate change due to change in the canopy (Figure 4). Overall, the percentage germination of seeds and growth performance of seedling were higher in the open canopy treatment compared to close canopy treatment. This suggest close canopy condition may act as a regulating factor for *R. serpentina* , as it generally prefers canopy gaps (Department of Plant Resources 2013; Dey and De 2010; Kunwar 2019). Therefore, canopy openness and closeness can significantly influence percentage seed germination and growth performance of *R. serpentina* .

Additionally, high temperatures in the open canopy treatment may also contribute to the increased seed germination rate and seedling growth performance compared to the close canopy treatment in polyhouse, as the temperature was reported to regulate percentage germination of seeds and growth performance of seedlings (Hussain and Jha 2014; Muneshwar 2015). Hussain and Jha (2014) reported an approximately three‐fold increase in percentage germination of *R. serpentina* , when the temperature was raised from 30°C to 35°C. Similarly, Muneshwar (2015) also documented higher seed germination percentage and improved growth performance of *R. serpentina* seedling grown at the higher temperature conditions. Thus, both open canopy and higher temperature in open canopy treatment may have enhanced the seed germination potentiality and seedling growth performance of *R. serpentina* in the polyhouse.

### 4.6 Effect of Invasion of *Mikania*

Apart from direct anthropogenic disturbances, the invasion of *Mikania* might have also affected the population dynamics of *R. serpentina* in the Jalthal forest. *Mikania* is known to decrease the density of associated native species (Ismail and Mah 1993; Kaur et al. 2012). *Mikania* degrades habitat quality and kills the native plant species through allelopathy, blocking sunlight, twinning, and smothering (Shrestha 2016; Yin et al. 2020; Zhang et al. 2004). A similar situation was observed in the highly disturbed and previously disturbed sites of the Jalthal forest, where *Mikania* had overtaken the native vegetation and completely overgrown other plants, forming mono‐specific stand on the ground and suppressing the growth of native species like *R. serpentina* (Chen et al. 2024; Shen et al. 2015; Zhang et al. 2004). The native plant community in the Jalthal forest faced serious threats due to this invasive plant species. Since both *R. serpentina* and *Mikania* prefer open areas or canopy gaps of the forest, the invasion of *Mikania* might have directly hampered the population performance of *R. serpentina* in the Jalthal forest through competition and habitat alteration (Kaur et al. 2012; Kunwar 2019). Additionally, anthropogenic activities increase the probability of invasion. Thus, the population of *R. serpentina* in the Jalthal forest is at risks if the current condition persists (Simberloff 2009).

The low percentage germination of seeds and growth performance of seedlings of *R. serpentina* from the close with‐mikania canopy type can be attributed to the negative effect of the invasion of *Mikania*, which is known to suppress the germination and growth of associated species (Shen et al. 2015; Srivastava et al. 2014; Xu et al. 2013). Allelochemicals released by *Mikania* alter the soil nutrients and the microbiome community, inhibiting seed germination rate and seedling growth of other native species and creating positive feedback loop, which promote further invasion (Ismail and Mah 1993; Saikia and Khan 2013; Shen et al. 2015; Xu et al. 2013; Yin et al. 2020; Zhang et al. 2004; Zhao et al. 2023). While we cannot pinpoint the exact reason behind the low percentage germination of seed and growth performance of seedling from the close with‐mikania canopy type, allelopathy likely plays a role. The soil from the close with‐mikania canopy type contained debris from *Mikania* roots and shoots, emitting allelochemicals during decomposition. These allelochemicals may have inhibited the percentage seed germination and growth performance of seedling of *R. serpentina* (Ismail and Chong 2002). Thus, the combined effect of invasive alien species and anthropogenic disturbance has led to decreased population dynamics of *R. serpentina* in the Jalthal forest. Therefore, immediate action to control the invasive species and human encroachment is necessary to mitigate this threat.

## 5 Conclusion

*Rauvolfia serpentina* is susceptible to anthropogenic disturbances and invasion of alien species. The combined effects of these factors may have significantly reduced population density, growth performance, and recruitment of *R. serpentina* , ultimately impacting its abundance, regeneration, and persistence in the Jalthal forest. Moreover, the poor seed germination rate of this species renders it even more vulnerable in anthropogenic landscapes. The seed germination rate and growth performance of *R. serpentina* seedlings in the polyhouse were likely affected by several factors, including canopy coverage, temperature variations, and allelopathic effect of *Mikania*. Immediate actions are necessary to ensure the long‐term persistence of *R. serpentina* in the Jalthal forest. Implementing a proper management and conservation plan that protects the remaining population of *R. serpentina* through controlling and monitoring invasion of alien species and regulating habitat alteration and degradation is crucial. Additionally, raising awareness among locals of Jalthal forest about sustainable collection methods of various non‐timber forest products (NTFPs), including *R. serpentina* is essential for conservation efforts in the Jalthal forest.

## Author Contributions

**Ajay Neupane:** conceptualization (equal), data curation (lead), formal analysis (lead), investigation (equal), methodology (equal), validation (equal), visualization (lead), writing – original draft (lead), writing – review and editing (equal). **Bikram Jnawali:** formal analysis (supporting), investigation (equal), visualization (supporting), writing – original draft (supporting), writing – review and editing (equal). **Suresh Kumar Ghimire:** conceptualization (equal), data curation (supporting), formal analysis (supporting), methodology (equal), validation (equal), writing – review and editing (equal).

## Conflicts of Interest

The authors declare no conflicts of interest.

## Supporting information


**Table S1:** Percentage plots experiencing certain type of disturbance on four different sites.

**Table S2:** Relationships between dependent variables (population parameters of *R. serpentina* ) and important explanatory variables based on multiple regressions (forward selection method was adopted).

**Table S3:** List of associated vascular plant species of *Rauvolfia serpentina*.

## Data Availability

The data that support the findings of this study are attached as Supporting Information in the journal portal or can also be downloaded from Dryad (https://doi.org/10.5061/dryad.vt4b8gv2q).

## References

[ece372731-bib-0001] Baral, S. R. , and P. P. Kurmi . 2006. A Compendium of Medicinal Plants in Nepal. Rachana Sharma.

[ece372731-bib-0002] Barik, S. K. , R. S. Tripathi , H. N. Pandey , and P. Rao . 1996. “Tree Regeneration in a Subtropical Humid Forest: Effect of Cultural Disturbance on Seed Production, Dispersal and Germination.” Journal of Applied Ecology 33, no. 6: 1551. 10.2307/2404793.

[ece372731-bib-0003] Bharali, S. , A. Paul , M. L. Khan , and L. B. Singha . 2012. “Impact of Altitude on Population Structure and Regeneration Status of Two Rhododendron Species in a Temperate Broad Leaved Forest of Arunachal Pradesh, India.” International Journal of Ecosystem 2, no. 1: 19–27. 10.5923/j.ije.20120201.04.

[ece372731-bib-0004] Bhattarai, K. P. 2017. “Enumeration of Flowering Plants in Tarai Sal (*Shorea robusta* Gaertn.) Forest of Jalthal, Eastern Nepal.” Journal of Plant Resources 15, no. 1: 14–20.

[ece372731-bib-0005] Bhattarai, N. K. , V. Tandon , and D. K. Ved . 2002. “Highlights and Outcomes of the Conservation Assessment and Management Plan (CAMP) Workshop, Pokhara, Nepal.” In Sharing Local and National Experience in Conservation of Medicinal and Aromatic Plants in South Asia, edited by N. K. Bhattarai and M. Karki , 46–53. IDRC/MAPPA Ministry of Forest and Soil Conservation, Kathmandu, Nepal; Institute of Forestry, Pokhara.

[ece372731-bib-0006] Chapagain, D. J. , H. Meilby , and S. K. Ghimire . 2019. “Plant Density and Life History Traits of *Aconitum Spicatum* in North‐Central Nepal: Effects of Elevation and Anthropogenic Disturbances.” PeerJ 7, no. 9: e7574. 10.7717/PEERJ.7574. 31565560 PMC6743441

[ece372731-bib-0007] Chaudhary, R. P. , Y. Uprety , S. P. Joshi , et al. 2015. Kanchenjunga Landscape Nepal: From Conservation and Development Perspectives. Ministry of Forest and Soil Conservation (MoFSC), Government of Nepal (GoN); Research Centre for Applied Science and Technology (RECAST), Tribhuvan University (TU); and International Center for Integrated Mountain Development (ICIMOD).

[ece372731-bib-0008] Chen, L. , M. Cai , Q. Zhang , et al. 2024. “Why Can *Mikania micrantha* Cover Trees Quickly During Invasion?” BMC Plant Biology 24, no. 1: 511. 10.1186/s12870-024-05210-5. 38844870 PMC11157800

[ece372731-bib-0009] Choudhary, J. 2003. “Standardization on Traditional Medicine.” Nature Biology 22, no. 3: 263–265.

[ece372731-bib-0010] Convention on Biological Diversity . 2009. “Plant Conservation Report: A Review of Progress in Implementing the Global Strategy of Plant Conservation (GSPC).” Secretariat of the Convention on Biological Diversity.

[ece372731-bib-0011] Department of Plant Resources . 2007. “Bulletin of the Department of Plant Resources no. 28 Medicinal Plants of Nepal (Revised).” Ministry of Forest and Soil Conservation, Government of Nepal.

[ece372731-bib-0012] Department of Plant Resources . 2012. “Plants of Nepal: Fact Sheet.” Ministry of Forests & Soil Conservation, Government of Nepal.

[ece372731-bib-0013] Department of Plant Resources . 2013. “Quality Standards, Good Agricultural and Collection Practice (GACP) of *Rauvolfia serpentina* (L.) Benth. *Ex kurz*.” Department of Plant Resources, Ministry of Forest and Soil Conservation, Goverment of Nepal.

[ece372731-bib-0014] Dey, A. , and J. N. De . 2010. “ *Rauvolfia serpentina* (L). Benth. Ex Kurz.–A Review.” Asian Journal of Plant Sciences 9, no. 6: 285–298. 10.3923/ajps.2010.285.298.

[ece372731-bib-0015] Dueñas, M.‐A. , H. J. Ruffhead , N. H. Wakefield , P. D. Roberts , D. J. Hemming , and H. Diaz‐Soltero . 2018. “The Role Played by Invasive Species in Interactions With Endangered and Threatened Species in the United States: A Systematic Review.” Biodiversity and Conservation 27, no. 12: 3171–3183. 10.1007/s10531-018-1595-x.

[ece372731-bib-0016] Dutta, P. K. , I. C. Chopra , and L. D. Kapoor . 1963. “Cultivation of *Rauvolfia serpentina* in India.” Economic Botany 17, no. 4: 243–251. 10.1007/BF02860133.

[ece372731-bib-0017] Endels, P. , H. Jacquemyn , R. Brys , and M. Hermy . 2007. “Genetic Erosion Explains Deviation From Demographic Response to Disturbance and Year Variation in Relic Populations of the Perennial *Primula vulgaris* .” Journal of Ecology 95, no. 5: 960–972. 10.1111/j.1365-2745.2007.01279.x.

[ece372731-bib-0018] Gaire, D. , L. Jiang , B. Adhikari , S. Bhattarai , and S. Panthi . 2022. “Predicting the Potential Distribution, Trade, and Conservation of *Rauvolfia serpentina* in Nepal.” Applied Ecology and Environmental Research 20, no. 6: 4999–5022. 10.15666/aeer/2006_49995022.

[ece372731-bib-0019] Ghimire, S. K. 2008. “Medicinal Plants in Nepal Himalayas: Current Issues, Sustainable Harvesting Knowledge Gaps and Research Priorities.” In Medicinal Plants in Nepal: An Anthology of Contemporary Research, edited by P. K. Jha , S. B. Karmacharya , M. K. Chhetri , C. B. Thapa , and B. B. Shrestha , 25–43. Ecological Society.

[ece372731-bib-0020] Ghimire, S. K. , O. Gimenez , R. Pradel , D. McKey , and Y. Aumeeruddy‐Thomas . 2007. “Demographic Variation and Population Viability in a Threatened Himalayan Medicinal and Aromatic Herb *Nardostachys grandiflora*: Matrix Modelling of Harvesting Effects in Two Contrasting Habitats.” Journal of Applied Ecology 45, no. 1: 41–51. 10.1111/J.1365-2664.2007.01375.X.

[ece372731-bib-0021] Ghimire, S. K. , D. McKey , and Y. Aumeeruddy‐Thomas . 2005. “Conservation of Himalayan Medicinal Plants: Harvesting Patterns and Ecology of Two Threatened Species, *Nardostachys grandiflora* DC. and *Neopicrorhiza scrophulariiflora* (Pennell) Hong.” Biological Conservation 124, no. 4: 463–475. 10.1016/j.biocon.2005.02.005.

[ece372731-bib-0022] Given, D. R. 1975. “Conservation of Rare and Threatened Plant Taxa in New Zealand–Some Principles.” Proceedings (New Zealand Society) 22: 1–6.

[ece372731-bib-0023] Gupta, P. K. 2001. Methods in Environmental Analysis of Water, Soil and Air. Second ed. Agrobios.

[ece372731-bib-0024] Hara, H. , W. T. Stearn , and L. H. J. Williams . 1979. Enumeration of the Flowering Plants of Nepal: Vol. Second. Trustees of British Museum (Natural History).

[ece372731-bib-0025] Hartnett, D. C. , and F. A. Bazzaz . 1985. “The Regulation of Leaf, Ramet and Genet Densities in Experimental Populations of the Rhizomatous Perennial *Solidago Canadensis* .” Journal of Ecology 73, no. 2: 429. 10.2307/2260485.

[ece372731-bib-0026] Hussain, A. , and D. Jha . 2014. “Seed Germination Improvement in Two Threatened Medicinal Plants.” Current Agriculture Research Journal 2, no. 2: 131–136. 10.12944/CARJ.2.2.10.

[ece372731-bib-0027] Ismail, B. S. , and T.‐V. Chong . 2002. “Effects of Aqueous Extracts and Decomposition of *Mikania micrantha* H.B.K. Debris on Selected Agronomic Crops.” Weed Biology and Management 2, no. 1: 31–38. 10.1046/j.1445-6664.2002.00045.x.

[ece372731-bib-0028] Ismail, B. S. , and L. S. Mah . 1993. “Effects of *Mikania micrantha* H.B.K. On Germination and Growth of Weed Species.” Plant and Soil 157, no. 1: 107–113. 10.1007/BF00038753.

[ece372731-bib-0029] Jolliffe, I. T. 2002. Principal Component Analysis. Second ed. Springer.

[ece372731-bib-0030] Joshi, K. K. , and S. D. Joshi . 2006. Medicinal and Aromatic Plants Used in Nepal, Tibet and Trans‐Himalayan Region. Author House.

[ece372731-bib-0031] Joshi, N. , K. Sharma (Dhakal) , and D. S. Saud . 2017. “Checklist of CITES Listed Flora of Nepal.” Department of Plant Resources, Ministry of Forests and Soil Conservation, Government of Nepal.

[ece372731-bib-0032] Kala, C. P. , and Y. Dubey . 2012. “Anthropogenic Disturbances and Status of Forest and Wildlife in the Dry Deciduous Forests of Chhattisgarh State in India.” Journal of Forestry Research 23, no. 1: 45–52. 10.1007/s11676-012-0219-7.

[ece372731-bib-0033] Kalibo, H. W. , and K. E. Medley . 2007. “Participatory Resource Mapping for Adaptive Collaborative Management at Mt. Kasigau, Kenya.” Landscape and Urban Planning 82, no. 3: 145–158. 10.1016/J.LANDURBPLAN.2007.02.005.

[ece372731-bib-0034] Kaur, R. , S. Malhotra , and Inderjit 2012. “Effects of Invasion of *Mikania micrantha* on Germination of Rice Seedlings, Plant Richness, Chemical Properties and Respiration of Soil.” Biology and Fertility of Soils 48, no. 4: 481–488. 10.1007/s00374-011-0645-2.

[ece372731-bib-0035] Khan, M. S. , M. M. Rahman , and M. A. Ali . 2001. Red Data Book of Vascular Plants of Bangladesh. Bangladesh National Herbarium.

[ece372731-bib-0036] Kunwar, B. B. 2019. “Establishing In Situ Gene Bank of *Rauvolfia serpentina* (L.) Benth ex Kurtz in Western Nepal With a Focus on Conservation and Sustainability.” Biodiversity International Journal 3, no. 4: 139–143. 10.15406/bij.2019.03.00138.

[ece372731-bib-0037] Li, W. , R. Tan , J. Wang , F. Du , and Y. Yang . 2013. “Effects of Anthropogenic Disturbance on Richness‐Dependent Stability in Napahai Plateau Wetland.” Chinese Science Bulletin 58, no. 33: 4120–4125. 10.1007/s11434-013-5954-4.

[ece372731-bib-0038] Łomnicki, A. 1980. “Regulation of Population Density due to Individual Differences and Patchy Environment.” Oikos 35, no. 2: 185. 10.2307/3544426.

[ece372731-bib-0039] Lowe, S. , M. Browne , S. Boudjelas , and M. DePoorter . 2000. 100 of the World's Worst Invasive Alien Species: A Selection From the Global Invasive Species Database. Invasive Species Specialist Group.

[ece372731-bib-0040] Maiti, S. , and K. A. Geetha . 2013. “Country Status Report on Medicinal and Aromatic Plants in India.” In Expert Consultation on Promotion of Medicinal and Aromatic Plants in the Asia‐Pacific Region: Proceedings, edited by R. Paroda , S. Dasgupta , B. Mal , S. P. Ghosh , and S. K. Pareek , 101–123. Asia‐Pacific Association of Agricultural Research Institutions (APAARI), Food and Agriculture Organization of the United Nations–Regional Office for Asia and the Pacific (FAO RAP).

[ece372731-bib-0041] Malik, D. , and S. C. Swain . 2018. “Influence of Growing Media and Seed Treatment on Seed Germination and Seedling Vigour of Sarpagandha (*Rauvolfia serpentina* (L.), Benth. Ex Kurz).” Internation Journal of Chemical Studies 6, no. 5: 3388–3392.

[ece372731-bib-0042] Ministry of Science and Technology (MOST), & Vietnam Academy of Science and Technology (VAST) . 2007. Vietnam Red Data Book: Part II. Plants (In Vietnamese), edited by T. B. Nguyen , D. L. Tran , and K. K. Nguyen , vol. 2. Science and Technology Publishing House.

[ece372731-bib-0043] Mittal, B. , M. Meenakshi , A. S. Anitha Sharma , and V. K. Gotheca . 2012. “Phytochemical & Pharmacological Activity of *Rauwolfia serpentina*–A Review.” International Journal of Ayurvedic and Herbal Medicine 2, no. 3: 427–434.

[ece372731-bib-0044] Mulliken, T. , and P. Crofton . 2008. Review of the Status, Harvest, Trade and Management of Seven Asian CITES‐Listed Medicinal and Aromatic Plant Species. Bundesamt für Naturschutz (BfN), Federal Agency for Nature Conservation. www.dnl‐online.de.

[ece372731-bib-0045] Muneshwar, B. R. 2015. Standarization of Seed Germination Testing Procedure in Sarpagandha (*Rauvolfia serpentina* Benth.) Mahatma Phule Krishi Vidyapeeth.

[ece372731-bib-0046] Nair, V. D. , R. P. D. Raj , R. Panneerselvam , and R. Gopi . 2014. “Assessment of Diversity Among Populations of *Rauvolfia serpentina* Benth. Ex. Kurtz. From Southern Western Ghats of India, Based on Chemical Profiling, Horticultural Traits and RAPD Analysis.” Fitoterapia 92: 46–60. 10.1016/j.fitote.2013.09.017. 24096162

[ece372731-bib-0047] Oksanen, J. , G. L. Simpson , F. G. Blanchet , et al. 2024. “*vegan*: Community Ecology Package (R Package Version 2.8–0).” https://vegandevs.github.io/vegan/.

[ece372731-bib-0100] Oosttermeijer, J. G. B. , S. H. Luitjen , Z. V. Klenova , and J. C. M. Den Nijs . 1998. “Relationships Between Population and Habitat Characteristics and Reproduction of the rare *Gentiana pneumonanthe* L.” Conservation Biology 12, no. 5: 1042–1056.

[ece372731-bib-0048] Panwar, G. S. , and S. K. Guru . 2012. “Influence of Gibberellic Acid and Seed Coat Removed on the Seed Germination Behavior of *Rauwolfia serpentina* L. Under Controlled Environment.” Journal of Non‐Timber Forest Products 19: 1–4.

[ece372731-bib-0049] Paul, D. , N. K. Paul , and P. K. Basu . 2008. “Seed Germination Response of *Rauvolfia serpentina* Benth. To Certain Physical and Chemical Treatments.” Journal of Biological Sciences 16: 129–131.

[ece372731-bib-0050] Pavlovic, N. B. 1994. “Disturbance‐Dependent Persistence of Rare Plants: Anthropogenic Impacts and Restoration Implications.” In Restoration of Endangered Species, edited by M. L. Bowles and C. J. Whelan , 159–193. Cambridge University Press. 10.1017/CBO9780511623325.010.

[ece372731-bib-0051] Phatak, R. S. , N. K. Hegde , P. M. Gangadharappa , and L. Hegde . 2017. “Effect of Seed Treatment on Germination in Sarpagandha (*Rauvolfia serpentina* Benth).” International Journal of Current Microbiology and Applied Sciences 6, no. 12: 135–140. 10.20546/ijcmas.2017.612.018.

[ece372731-bib-0052] Plants of the World Online . 2024. *Rauvolfia serpentina* (L.) Benth. ex Kurz. Royal Botanic Gardens.

[ece372731-bib-0053] Polunin, O. , and A. Stainton . 1984. Flowers of Himalaya. Oxford University Press.

[ece372731-bib-0054] Poudyal, B. K. 2013. “Regeneration of Hill Sal and Plant Diversity in Community Forest: A Case Study From Pragatisil Community Forest in Kaski District, Western Nepal.” Banko Jankari 23, no. 2: 37–43.

[ece372731-bib-0055] Primack, R. B. 2010. Essentials of Conservation Biology. Fifth ed. Macmillan Science.

[ece372731-bib-0056] Pulliam, H. R. , and B. J. Danielson . 1991. “Sources, Sinks, and Habitat Selection: A Landscape Perspective on Population Dynamics.” American Naturalist 137: S50–S66. 10.1086/285139.

[ece372731-bib-0057] R Core Team . 2024. R: A Language and Environment for Statistical Computing. R Foundation for Statistical Computing. https://www.R‐project.org.

[ece372731-bib-0058] Rabinowitz, D. 1981. “Seven Forms of Rarity.” In The Biological Aspects of Rare Plant Conservation, edited by H. Synge , 205–217. John Wiley & Sons, Ltd.

[ece372731-bib-0059] Rahman, M. , A. Nishat , and H. Vacik . 2009. “Anthropogenic Disturbances and Plant Diversity of the Madhupur Sal Forests (*Shorea robusta* C.F. Gaertn) of Bangladesh.” International Journal of Biodiversity Science & Management 5, no. 3: 162–173. 10.1080/17451590903236741.

[ece372731-bib-0060] Regmi, A. R. , H. L. Shrestha , D. Mahatara , and B. Bhattarai . 2023. “Mapping of Invasive Plant Species in Jalthal Forest of Nepal Using High Resolution Remote Sensing Data.” Indian Journal of Weed Science 55, no. 4: 437–443. 10.5958/0974-8164.2023.00079.5.

[ece372731-bib-0061] Ricciardi, A. , T. M. Blackburn , J. T. Carlton , et al. 2017. “Invasion Science: A Horizon Scan of Emerging Challenges and Opportunities.” Trends in Ecology & Evolution 32, no. 6: 464–474. 10.1016/j.tree.2017.03.007. 28395941

[ece372731-bib-0062] Ricciardi, A. , R. J. Neves , and J. B. Rasmussen . 1998. “Impending Extinctions of North American Freshwater Mussels (*Unionoida*) Following the Zebra Mussel ( *Dreissena polymorpha* ) Invasion.” Journal of Animal Ecology 67, no. 4: 613–619. 10.1046/j.1365-2656.1998.00220.x.

[ece372731-bib-0063] Saikia, P. , and M. L. Khan . 2013. “Population Structure and Regeneration Status of *Aquilaria malaccensis* Lam. In Homegardens of Upper Assam, Northeast India.” Tropical Ecology 54, no. 1: 1–13.

[ece372731-bib-0064] Samanhudi, S. , E. Purwanto , S. Sulandjari , and A. S. Setiyaningsih . 2017. “Microclimate Modification Through Shading and Watering Frequency Treatments as Efforts for Ex Situ Conservation of Pule Pandak (*Rauvolfia serpentina*).” Asian Journal of Agriculture 1, no. 1: 35–39. 10.13057/asianjagric/g010107.

[ece372731-bib-0065] Saxena, A. K. , and J. S. Singh . 1984. “Tree Population Structure of Certain Himalayan Forest Associations and Implications Concerning Their Future Composition.” Vegetatio 58, no. 2: 61–69. 10.1007/BF00044928.

[ece372731-bib-0066] Schemske, D. W. , B. C. Husband , M. H. Ruckelshaus , C. Goodwillie , I. M. Parker , and J. G. Bishop . 1994. “Evaluating Approaches to the Conservation of Rare and Endangered Plants.” Ecology 75, no. 3: 584–606. 10.2307/1941718.

[ece372731-bib-0067] Sharma, L. N. , Y. B. Poudel , R. Pun Magar , et al. 2024. Jalthal Biodiversity Approach: Conservation of Plant Diversity With Emphasis on Rare and Threatened Trees in Jalthal Remnant Forest. ForestAction Nepal.

[ece372731-bib-0068] Sharma, L. N. , S. R. Tamang , Y. B. Poudel , et al. 2021. “Biodiversity Beyond Protected Areas: Gaps and Opportunities in Community Forest.” Journal of Forest and Livelihood 20, no. 1: 45–61.

[ece372731-bib-0069] Sharma, N. , and S. Tyagi . 2015. “Effect of Plant Growth Regulators (Indoles) on Germination Percentage and Seedling Growth of *Rauvolfia serpentina* (L.) Benth. Ex Kurz.” International Journal of Science and Research 4, no. 5: 1234–1238.

[ece372731-bib-0070] Sharma, P. , M. Roy , and B. Roy . 2022. “A Review on Influence of Floral Biology, Pollination Efficiency and Conservation Strategies of Endangered Medicinal Plant, *Rauvolfia serpentina* (L.) Benth. Ex Kurz.” Annals of Phytomedicine: An International Journal 11, no. 1: 86–98. 10.54085/ap.2022.11.1.9.

[ece372731-bib-0071] Sharma, R. 2004. Agro Techniques of Medicinal Plants. Daya Publishing House.

[ece372731-bib-0072] Shen, S. , G. Xu , D. R. Clements , et al. 2015. “Suppression of the Invasive Plant Mile‐a‐Minute (*Mikania micrantha*) by Local Crop Sweet Potato (*Ipomoea batatas*) by Means of Higher Growth Rate and Competition for Soil Nutrients.” BMC Ecology 15, no. 1: 1. 10.1186/s12898-014-0033-5. 25626963 PMC4325956

[ece372731-bib-0073] Shrestha, B. B. 2016. “Invasive Plant Species in Nepal.” In Frontiers of Botany, edited by P. K. Jha , M. Siwakoti , and S. Rajbhandary , 269–284. Central Department of Botany.

[ece372731-bib-0074] Shrestha, B. B. , A. S. Poudel , and M. Pandey . 2024. “Plant Invasions in Nepal: What we Do Not Know?” In Flora and Vegetation of Nepal, edited by M. B. Rokaya and S. R. Sigdel , vol. 19, 333–360. Plant and Vegetation. Springer. 10.1007/978-3-031-50702-1_13.

[ece372731-bib-0075] Shrestha, K. K. , P. Bhandari , and S. Bhattarai . 2022. Plant of Nepal (Gymnosperm and Angiosperms). Heritage Publishers & Distributors Pvt. Ltd.

[ece372731-bib-0076] Shrestha, L. J. , M. P. Devkota , and B. K. Sharma . 2016. “Tree Regeneration in Sacred Groves of Kathmandu Valley, Nepal.” Ecoprint: An International Journal of Ecology 22: 29–38. 10.3126/eco.v22i0.15468.

[ece372731-bib-0077] Simberloff, D. 2009. “The Role of Propagule Pressure in Biological Invasions.” Annual Review of Ecology, Evolution, and Systematics 40, no. 1: 81–102. 10.1146/annurev.ecolsys.110308.120304.

[ece372731-bib-0078] Singh, P. , P. Singh , S. Sahu , et al. 2017. “Ecological and Conservation Study of Herbs in Mukundpur Forest Area, Satna District, Madhya Pradesh.” Journal of Pharmacognosy and Phytochemistry 6, no. 4: 544–549. https://www.phytojournal.com/archives/2017.v6.i4.1391/ecological‐and‐conservation‐study‐of‐herbs‐in‐mukundpur‐forest‐area‐satna‐district‐madhya‐pradesh.

[ece372731-bib-0079] Šmilauer, P. , and J. Lepš . 2014. Multivariate Analysis of Ecological Data Using Canoco 5, Second Edition. Cambridge University Press.

[ece372731-bib-0080] Sooraj, N. P. , R. Jaishanker , C. R. Sajeev , V. S. Kumar , D. Lijimol , and J. Ammini . 2020. “Influence of Forest Canopy Gaps on Establishment of *Mikania micrantha* Kunth, an Invasive Plant, in a Tropical Forest in Southern Western Ghats, India.” Applied Ecology and Environmental Sciences 8, no. 5: 199–206.

[ece372731-bib-0081] Srivastava, S. , A. Dvivedi , and R. P. Shukla . 2014. “Invasive Alien Species of Terrestrial Vegetation of North‐Eastern Uttar Pradesh.” International Journal of Forestry Research 2014: 1–9. 10.1155/2014/959875.

[ece372731-bib-0082] Ticktin, T. 2004. “The Ecological Implications of Harvesting Non‐Timber Forest Products.” Journal of Applied Ecology 41, no. 1: 11–21. 10.1111/j.1365-2664.2004.00859.x.

[ece372731-bib-0083] Tiwari, A. K. , P. P. S. Verma , D. Kumar , et al. 2021. “Germination Behavior of *Rauvolfia serpentina* (L.) Seeds With the Use of Different Seed Treatments.” Indian Journal of Agricultural Research 57, no. 5: 671–675. 10.18805/IJARe.A-5624.

[ece372731-bib-0084] Tiwari, S. , M. Siwakoti , B. Adhikari , and K. Subedi . 2005. An Inventory and Assessment of Invasive Alien Plant Species of Nepal. IUCN ‐ The World Conservation Union.

[ece372731-bib-0085] Tonnabel, J. , F. M. Schurr , F. Boucher , et al. 2018. “Life‐History Traits Evolved Jointly With Climatic Niche and Disturbance Regime in the Genus *Leucadendron* (Proteaceae).” American Naturalist 191, no. 2: 220–234. 10.1086/695283. 29351009

[ece372731-bib-0086] Totland, O. , and J. Nyléhn . 1998. “Assessment of the Effects of Environmental Change on the Performance and Density of *Bistorta vivipara*: The Use of Multivariate Analysis and Experimental Manipulation.” Journal of Ecology 86, no. 6: 989–998. 10.1046/J.1365-2745.1998.00318.X.

[ece372731-bib-0087] Trivedi, M. P. , and R. Kumari . 2009. “Germination Potential of *Rauvolfia serpentina* in Varying Environmental Regimes.” Journal of the Indian Botanical Society 88, no. 3–4: 58–61.

[ece372731-bib-0088] Vasisht, K. , N. Sharma , and M. Karan . 2016. “Current Perspective in the International Trade of Medicinal Plants Material: An Update.” Current Pharmaceutical Design 22, no. 27: 4288–4336. 10.2174/1381612822666160607070736. 27281331

[ece372731-bib-0089] Watson, L. E. , G. E. Uno , N. A. McCarty , and A. B. Kornkven . 1994. “Conservation Biology of a Rare Plant Species, *Eriocaulon kornickianum* (Eriocaulaceae).” American Journal of Botany 81, no. 8: 980–986. 10.1002/j.1537-2197.1994.tb15585.x.

[ece372731-bib-0090] Xu, Q. , H. Xie , H. Xiao , L. Lin , and X. Wei . 2013. “Two New Ent‐Kaurene Diterpene Glucosides From the Roots of *Mikania micrantha* .” Phytochemistry Letters 6, no. 3: 425–428. 10.1016/j.phytol.2013.05.007.

[ece372731-bib-0091] Yin, L. , B. Liu , H. Wang , et al. 2020. “The Rhizosphere Microbiome of *Mikania micrantha* Provides Insight Into Adaptation and Invasion.” Frontiers in Microbiology 11: 1462. 10.3389/fmicb.2020.01462. 32733410 PMC7359623

[ece372731-bib-0092] Zhang, L. Y. , W. H. Ye , H. L. Cao , and H. L. Feng . 2004. “ *Mikania micrantha* H.B.K. In China‐An Overview.” Weed Research 44, no. 1: 42–49. 10.1111/j.1365-3180.2003.00371.x.

[ece372731-bib-0093] Zhao, P. , B. Liu , H. Zhao , Z. Lei , and T. Zhou . 2023. “Significant Changes in Soil Microbial Community Structure and Metabolic Function After *Mikania micrantha* Invasion.” Scientific Reports 13, no. 1: 1141. 10.1038/s41598-023-27851-6. 36670134 PMC9860029

