# Supplementary material for: Anthropogenic Disturbances and Invasion of Mikania micrantha Threaten Rauvolfia serpentina Populations in Nepal
Source: Ecol Evol. 2025 Dec 22;15(12):e72731. doi: 10.1002/ece3.72731 (PMC12720017; doi:10.1002/ece3.72731)
Supplement: Supplementary file 1 — Table S1: Percentage plots experiencing certain type of disturbance on four different sites. [file ECE3-15-e72731-s003.docx]

# Percentage plots experiencing certain type of disturbance on four different sites.

| **Site** | **Disturbance type** | **% plot experiencing certain type of disturbance** | | | | |
| --- | --- | --- | --- | --- | --- | --- |
|  |  | **0** | **1** | **2** | **3** | **4** |
| JHJ | Animal Droppings | 22.5 | 62.5 | 5 | 5 | 5 |
| Highly | Grazing | 40 | 50 | 7.5 | 0 | 2.5 |
| Disturbed | Trampling | 10 | 40 | 25 | 20 | 5 |
|  | Harvesting | 20 | 32.5 | 17.5 | 17.5 | 12.5 |
|  | Fire | 85 | 15 | 0 | 0 | 0 |
|  | Overall | 35.5 | 40 | 11 | 8.5 | 5 |
| LMD | Animal Droppings | 27.5 | 72.5 | 0 | 0 | 0 |
| Moderately | Grazing | 57.5 | 35 | 7.5 | 0 | 0 |
| Disturbed | Trampling | 45 | 32.5 | 7.5 | 7.5 | 7.5 |
|  | Harvesting | 60 | 7.5 | 7.5 | 17.5 | 7.5 |
|  | Fire | 95 | 5 | 0 | 0 | 0 |
|  | Overall | 57 | 30.5 | 4.5 | 5 | 3 |
| CHA | Animal Droppings | 80 | 7.5 | 12.5 | 0 | 0 |
| Undisturbed | Grazing | 95 | 5 | 0 | 0 | 0 |
|  | Trampling | 20 | 80 | 0 | 0 | 0 |
|  | Harvesting | 77.5 | 17.5 | 5 | 0 | 0 |
|  | Fire | 100 | 0 | 0 | 0 | 0 |
|  | Overall | 74.5 | 22 | 3.5 | 0 | 0 |
| BLD | Animal Droppings | 10 | 72.5 | 17.5 | 0 | 0 |
| Previously | Grazing | 80 | 10 | 10 | 0 | 0 |
| Disturbed | Trampling | 80 | 10 | 10 | 0 | 0 |
|  | Harvesting | 100 | 0 | 0 | 0 | 0 |
|  | Fire | 100 | 0 | 0 | 0 | 0 |
|  | Overall | 74 | 18.5 | 7.5 | 0 | 0 |
