# Supplementary material for: Anthropogenic Disturbances and Invasion of Mikania micrantha Threaten Rauvolfia serpentina Populations in Nepal
Source: Ecol Evol. 2025 Dec 22;15(12):e72731. doi: 10.1002/ece3.72731 (PMC12720017; doi:10.1002/ece3.72731)
Supplement: Supplementary file 2 — Table S2: Relationships between dependent variables (population parameters of R. serpentina ) and important explanatory variables based on multiple regressions (forward selection method was adopted). [file ECE3-15-e72731-s001.docx]

# Relationships between dependent variables (population parameters of *R. serpentina*) and important explanatory variables based on multiple regressions (forward selection method was adopted).

| Dependent variable | Important explanatory variable | *R^2^* | *β* | *F* | *p* |
| --- | --- | --- | --- | --- | --- |
| Seedling density | Litter cover | 0.075 | -0.24 | 6.38 | 0.002 |
|  | Grass cover |  | -0.21 |  |  |
| Juvenile density | Litter cover | 0.064 | -0.23 | 6.42 | 0.002 |
|  | Grass cover |  | -0.21 |  |  |
| Adult vegetative density | Harvesting and grazing | 0.040 | -0.21 | 7.60 | 0.007 |
| Adult reproductive density | Harvesting and grazing | 0.051 | -0.24 | 9.525 | 0.002 |
| Total population density | Harvesting and grazing | 0.21 | -0.17 | 14.86 | <0.001 |
|  | Grass cover |  | -0.30 |  |  |
|  |  |  | -0.311 |  |  |
| No. of main branch per individual | Harvesting and grazing | 0.053 | -0.17 | 5.43 | 0.005 |
|  | Litter cover |  | -0.16 |  |  |
| Adult height | Harvesting and grazing | 0.054 | -0.24 | 10.12 | 0.002 |
| Adult stem diameter | Harvesting and grazing | 0.061 | -0.26 | 11.34 | 0.001 |
| Adult canopy diameter | Harvesting and grazing | 0.072 | -0.28 | 13.41 | <0.001 |
| Total reproductive output per individual | Harvesting and grazing | 0.043 | -0.22 | 8.14 | 0.005 |
