# Supplementary material for: Anthropogenic Disturbances and Invasion of Mikania micrantha Threaten Rauvolfia serpentina Populations in Nepal
Source: Ecol Evol. 2025 Dec 22;15(12):e72731. doi: 10.1002/ece3.72731 (PMC12720017; doi:10.1002/ece3.72731)
Supplement: Supplementary file 3 — Table S3: List of associated vascular plant species of Rauvolfia serpentina. [file ECE3-15-e72731-s002.docx]

List of associated vascular plant species of *Rauvolfia serpentina.*

| Species Name | Abbrevation | Family |
| --- | --- | --- |
| *Terminalia alata* Heyne ex. Roth. | Teal | Combretaceae |
| *Shorea robusta* Gaertn. | Shro | Dipterocarpaceae |
| *Terminalia bellirica* (Gaertn.) Roxb. | Tebe | Combretaceae |
| *Flemingia strobilifera* (L.) W.T. Aiton | Flst | Leguminosae |
| *Achyranthes asper*a L | Acas | Amaranthaceae |
| *Digitaria ciliaris* (Retz.) Koeler | Dici | Poaceae |
| *Cissus repens* Lam. | Cire | Vitaceae |
| *Sida cordata* ( Burm. f.) Bross. Waalk. | Sico | Malvaceae |
| *Piper longum* L. | Pilo | Piperaceae |
| *Leea aequata* L. | Leae | Vitaceae |
| *Chromolaena odorata* (L.) R.M. King & H. Robb. | Chod | Compositae |
| *Clerodendrum japonicum* (Thunb.) Sweet | Clja | Lamiaceae |
| *Barleria cristata* L. | Baci | Acanthaceae |
| *Hellenia speciosa (J.Koenig) S.R.Dutta* | Chsp | Costaceae |
| *Pouzolzia zeylanica* (L.) Benn. & R.Br. | Poze | Urticaceae |
| *Axonopus compressus*(Sw.) P.Beauv. | Axco | Poaceae |
| *Oplismenus burmanni* (Retz.) P.Beauv. | Opbu | Poaceae |
| *Casearia graveolens* Dalzell | Cagr | Buxaceae |
| *Lygodium flexuosum* (L.) Sw. | Lyfl | Lygodiacaeae |
| *Garuga pinnata* Roxb. | Gapi | Burseraceae |
| *Lagerstroemia parviflora* Roxb. | Lapa | Lythraceae |
| *Millettia extensa* (Benth.) Baker | Miex | Fabaceae |
| *Pteris vittata* L. | Ptvi | Pteridaceae |
| *Dioscorea deltoidea* Wall. ex Griseb. | Dide | Dioscoreaceae |
| *Antidesma bunius* (L.) Spreng | Anbu | Phyllanthaceae |
| *Cyperus cyperinus* (Retz.) Suringar | Cycy | Cyperaceae |
| *Aegle marmelos* (L.) Corrêa | Aema | Rutaceae |
| *Murraya koenigii* (L.) Spreng. | Muko | Rutaceae |
| *Smilax ovalifolia* Roxb. ex. D. Don | Smov | Smilaceae |
| *Adiantum philippense* L. | Adph | Pteridaceae |
| *Urochloa ramosa* (L.) T.Q.Nguyen | Urra | Poaceae |
| *Ageratina adenophora* (Spreng.) R.M. King & H.Robb. | Agad | Compositae |
| *Mallotus philippensis*(Lam.) Müll.Arg. | Maph | Euphorbiaceae |
| *Leea asiatica* (L.) Ridsdale | Leas | Vitaceae |
| *Ampelocissus latifolia* (Roxb.) Planch | Amla | Vitaceae |
| *Phanera vahlii*(Wight & Arn.) Benth. | Phva | Leguminosae |
| *Dioscorea bulbifera* L. | Dibu | Dioscoreaceae |
| *Panicum repens* L. | Pare | Poaceae |
| *Uncaria sessilifrutcus* Roxb. | Unse | Rubiaceae |
| *Phyllanthus amarus* Schumach & Thonn. | Pham | Phyllanthaceae |
| *Murdannia nudiflora* (L.) Brenan | Munu | Commelinaceae |
| *Adina cordifolia* Benth. & Hook. | Adco | Rubiaceae |
| *Schleichera oleosa* Lour. Merr. | Scol | Sapindaceae |
| *Curculigo orchioides* Gaertn. | Cuor | Hypoxidaceae |
| *Breynia vitis-idaea* (Burm.f.) C.E.C. Fisch. | Brvi | Phyllanthaceae |
| *Paspalum notatum* Flüggé | Pano | Poaceae |
| *Globba clarkei* Baker | Glcl | Zingiberaceae |
| *Sida rhombifolia* L. | Sirh | Malvaceae |
| *Commelina benghalensis* L. | Cobe | Commelinaceae |
| *Thelypteris dentata* (Forssk.) E.P.St.John | Thde | Thelypteridaceae |
| *Drymaria cordata* (L.) Willd.ex Roem. & Schult. | Drco | Caryophyllaceae |
| *Oxalis corniculata* L. | Oxco | Oxalidaceae |
| *Ageratum conyzoides* (L.) L. | Agco | Compositae |
| *Pennisetum glaucam* (L.) R.Br. | Pegl | Poaceae |
| *Senna tora* (L.) Roxb. | Seto | Leguminosae |
| *Setaria parviflora* (Poir.) M.Kerguelen | Sepa | Poaceae |
| *Cynodon dactylon* (L.) Pers. | Cyda | Poaceae |
| *Crassocephalum crepidioides* (Benth.) S.Moore | Crcr | Compositae |
| *Melastoma malabathricum* L. | Mema | Melastomataceae |
| *Imperata cylindrica* (L.) Raeusch. | Imcy | Poaceae |
| *Centella asiatica* (L.) Urb. | Ceas | Apiaceae |
| *Eleusine indica* (L.) Gaertn. | Elin | Poaceae |
| *Stephania glabra* (Roxb.) Miers. | Stgl | Menispermaceae |
| *Alternanthera sessilis* (L.) R.Br. ex DC | Alse | Amaranthaceae |
| *Chrysopogon aciculatus* (Retz.) Trin. | Chac | Poaceae |
| *Clerodendrum infortunatum* L. | Clin | Lamiaceae |
